# The Sure Foundation

**Published:** 1889-10-19

**Authors:** 


### Words of Consolation.

### THE SURE FOUNDATION.

*(For Reading to the Sick.)*

Nothing so much prevents peace and happiness reigning in our hearts as a feeling of instability. We go on from day to day with our work and our pleasures. Things are fairly prosperous, no particular anxiety troubles us, either about ourselves or our loved ones, and yet behind this seeming calm lurks a sense of uncertainty, a want of something, we know not what, a desire for rest, a presentiment that sorrows will come sooner or later. Our Heavenly Father, in kindness and wisdom, allows these thoughts to beset us, lest we should build our spiritual house on the sandy foundation of pleasure, or our own goodness, or the numerous temptations which lead us away from Christ, so that when the winds and storms of adversity beat upon us our faith should fail, and we fall away from the truth.

We are speaking now to those who have been planted by baptism in the Church, whose foundations are in the Rock, our Lord Jesus Christ, the Son of God. He who holds fast to this faith shall never fall; his house is built on the Rock of our Salvation: it shall never be removed.

"The God of our life, the Hope of every nation" will keep all that put their trust in Him. When thirsty and fainting in the desert of life He will bring water out of the stony rock to refresh us as He did the Israelites of old, and St. Paul tells us that rock was Christ.

In our battles with the world, the flesh, and the devil we can have confidence, for He is our Rock and our Fortress, our Defender in whom we will trust to shield us from the fiery darts of the evil one. Let us in all distresses cry "Lead me to the Rock that is higher than I, for Thou has been my shelter and a strong tower from the enemy." The Christian has, perhaps, let the cares and pleasures of life dull his vision, and he cannot see the reality of Christ being the only hope of salvation, and sorely needs the assurance of His grace.

Turn again, then, unto the stronghold, O restless soul, pray with all thine heart for His comfort and help. Hide thyself in the Rock of Ages which is always ready to shelter thee. Keep thy dear Lord ever before thee as thy Saviour and thy Guide, and thou shalt be upheld by the might of His strength.

But there are many who have never brought home to themselves their need of a Saviour; they know little of religion, and care less for it. Year by year they drift away farther from the Rock on to the quicksands of sin and pleasure. The sands are dry and smooth, the air soft and balmy, they sport on unmindful of the rising tide or the lowering clouds around; they make their home where they find their amusement. Then the storm bursts, the angry waves are beating around them, and the treacherous foundation is sinking away or drawing them in to their destruction. Will they fly to that Rock which alone can save them now their hour of suffering has arrived? Can they now be saved? Yes, thanks to God for His unspeakable mercy, there is hope for the most guilty. Only fling away the darling sin; fall on your knees in penitence; show your dear Lord your poverty, your weakness, your misery; ask in humble faith that He will shelter you from temptation, and your prayer shall be granted. He will clothe your nakedness with righteousness, having first cleansed you with His precious blood; He will feed you with the bread of life; He will give you to drink of living waters, and will guide your halting steps into the way of peace.

